# Susceptibility to type 1 diabetes conferred by the *PTPN22 *C1858T polymorphism in the Spanish population

**DOI:** 10.1186/1471-2350-8-54

**Published:** 2007-08-13

**Authors:** Jose Luis Santiago, Alfonso Martínez, Hermenegildo de la Calle, Miguel Fernández-Arquero, M Ángeles Figueredo, Emilio G de la Concha, Elena Urcelay

**Affiliations:** 1 Immunology Department, Hospital Universitario San Carlos, Madrid, Spain; 2 Endocrinology Department, Hospital Ramón y Cajal, Madrid, Spain

## Abstract

**Background:**

The protein tyrosine phosphatase N22 gene (*PTPN22*) encodes a lymphoid-specific phosphatase (LYP) which is an important downregulator of T cell activation. A *PTPN22 *polymorphism, C1858T, was found associated with type 1 diabetes (T1D) in different Caucasian populations. In this study, we aimed at confirming the role of this variant in T1D predisposition in the Spanish population.

**Methods:**

A case-control was performed with 316 Spanish white T1D patients consecutively recruited and 554 healthy controls, all of them from the Madrid area. The *PTPN22 *C1858T SNP was genotyped in both patients and controls using a TaqMan Assay in a 7900 HT Fast Real-Time PCR System.

**Results:**

We replicated for the first time in a Spanish population the association of the 1858T allele with an increased risk for developing T1D [carriers of allele T vs. CC: OR (95%) = 1.73 (1.17–2.54); p = 0.004]. Furthermore, this allele showed a significant association in female patients with diabetes onset before age 16 years [carriers of allele T vs. CC: OR (95%) = 2.95 (1.45–6.01), female patients vs female controls p = 0.0009]. No other association in specific subgroups stratified for gender, HLA susceptibility or age at onset were observed.

**Conclusion:**

Our results provide evidence that the *PTPN22 *1858T allele is a T1D susceptibility factor also in the Spanish population and it might play a different role in susceptibility to T1D according to gender in early-onset T1D patients.

## Background

Type 1 diabetes (T1D) is an autoimmune disorder resulting from destruction of the insulin-producing β cells of the pancreas. T1D is a complex trait with both genetic and environmental factors contributing to the etiology of this disease. Several susceptibility loci involved in disease development have been identified and were consistently replicated in independent populations [1]. These efforts contribute to a better definition of the molecular pathways leading to increased T1D risk and this knowledge, in turn, may help in understanding the genetic basis of the disease. The MHC class II, the CTLA4 and the *PTPN22 *loci have all been proved important in the pathogenesis of autoimmunity globally considered, whereas the insulin gene is a disease-specific T1D predisposition locus. Most of the new general susceptibility loci identified in the past few years have a clear role in the modulation of T cell development and activation, indicating that common biological pathways may be implicated in the etiology of different autoimmune diseases.

The role of a functional polymorphism of the *PTPN22 *gene, C1858T, in T1D susceptibility is well established. Bottini et al were the first to show that the minor allele of this variant confers predisposition to T1D using a case-control study with European American and Sardinian cohorts [2]. The allelic variation of the *PTPN22 *gene, 1858T, has been found associated with several autoimmune disorders: rheumatoid arthritis [3-7], systemic lupus erythematosus [4], Wegener's granulomatosis [8] and myasthenia gravis [9] among them, but no association was found with either multiple sclerosis [10] or celiac disease [11]. Confirmation of the *PTPN22 *association with T1D was performed in several independent populations [12-19]. Recently, a study described for the first time the sex-specific association of this polymorphism with T1D in Germany [20]. Gender-dependent association of PTPN22 has been described with rheumatoid arthritis[21] and with psoriatic arthritis[22], showing a predominant effect in male in both cases. The purpose of the present study was to verify the sex-biased association, as it might underlie a gender-dependent mechanism involved in disease pathogenesis.

## Methods

### Patients and controls

We studied 316 white unrelated Spanish T1D patients (159 women and 157 men) diagnosed according to the criteria of the American Diabetes Association (ADA) and 554 healthy controls recruited among blood donors from the Madrid area (therefore with an age ranging from 18–60 years). The gender distribution in our control group was: 51% females and 49% males; in 168 anonymous blood donors gender could not be ascertained.

The age at onset for the consecutively recruited T1D patients range from 1 to 55 years old (mean: 17.3 ± 10.0 and median age at onset: 15 years). All subjects were insulin-dependent at the time of the study. Informed consent was obtained from all the subjects included in the study, which was approved by the Ethics Committee of the Hospital Clínico San Carlos.

### Genotyping

Patients and controls were genotyped for the *PTPN22 *C1858T SNP (rs2476601) using a TaqMan Assay by Design in a 7900 HT Fast Real-Time PCR System (Applied Biosystem Foster City, CA, USA). HLA typing was performed as described by Urcelay et al [23].

### Statistical analysis

Differences in allele and genotype frequencies were calculated by Chi-square, or Fisher's exact test when necessary. Associations were estimated by the odds ratio (OR) with 95% confidence interval (CI). Statistical analysis used Epi Info v. 6.02 (CDC Atlanta USA). No deviations from Hardy-Weinberg equilibrium were observed for genotypes of this polymorphism in our population.

## Results

The case-control study showed that the distribution of the *PTPN22 *C1858T alleles in the Spanish population was, as expected, significantly different between patients and controls (Table 1). The minor allele 1858T confers susceptibility to T1D, as already described in other white populations. Based on the previously reported sex-specific association found in Germany [20], we tested for association in our cohorts after stratifying according to gender. No significant difference was detected between female and male T1D patients (Table 1).

**Table 1:** Analysis of the *PTPN22 *C1858T variant in the Spanish cohort

	Genotypes (%)			Alleles (%)	
Subjects	CC	CT	TT	C	T
Controls (n = 554)	483 (87.2)	68 (12.3)	3 (0.5)	1034 (93.3)	74 (6.7)
Female (n = 197)	172 (87.3)	23 (11.7)	2 (1.0)	367 (93.1)	27 (6.9)
Male (n = 189)	168 (89.9)	20 (10.6)	1 (0.5)	356 (94.2)	22 (5.8)
					
Cases (n = 316)	252 (79.7)	59 (18.7)	5 (1.6)	563 (89.1)	69 (10.9)
Female (n = 159)	122 (76.7)	33 (20.8)	4 (2.5)	277 (87.1)	41 (12.9)
Male (n = 157)	130 (82.8)	26 (16.1)	1 (0.6)	286 (91.1)	28 (8.9)

Patients vs. controls:

T vs. C; OR (95%) = 1.71 (1.20–2.45); p = 0.002

(TT+CT) vs. CC; OR (95%) = 1.73 (1.17–2.54); p = 0.004

In order to study the effect of age at onset on the distribution of genotypes of this *PTPN22 *polymorphism, we divided our patients in two groups, setting the median age at onset in our cohort (15 years) as the cut-off value. We selected median instead of mean age, because it is a better central statistic for highly skewed distributions, as T1D age at diagnosis is, and it maximizes the statistical power. We have age at disease onset data for 281 patients and the distribution of the 1858T allele was no significantly different between both groups: carriers of allele 1858T in younger patients vs. patients older than 15 years [31/141 vs. 28/140 (OR = 1.13; p = 0.68)].

However, when we considered both age and gender stratifications simultaneously (Table 2), in the pediatric-onset cohort (younger than 16 years old) the frequency of *PTPN22 *1858T allele was significantly increased in diabetic females, which is the only group significantly different from controls (carriers of 1858T, diabetic vs. healthy females: OR (95%) = 2.95 (1.45–6.01); p = 0.0009).

**Table 2:** Gender and age distribution of the *PTPN22 *C1858T polymorphism in Spanish T1D patients

	Genotypes (%)			T1D vs. Controls (carrier of T vs CC)	
	
Subjects	CC	CT	TT	OR CI (95%)	p
Female T1D ≤ 15 (n = 70)	49 (70.0)	19 (27.1)	2 (2.9)	2.92 (1.59–5.33)	0.0001
Male T1D ≤ 15 (n = 71)	61 (85.9)	10 (14.1)	0	1.12 (0.51–2.37)	0.76
Female T1D > 15 (n = 70)	56 (80.0)	12 (17.1)	2 (2.9)	1.70 (0.85–3.34)	0.10
Male T1D > 15 (n = 70)	56 (80.0)	13 (18.6)	1 (1.4)	1.70 (0.85–3.34)	0.10
					
Controls	483 (87.2)	68 (12.3)	3 (0.5)		

Female T1D ≤ 15 vs. Male T1D ≤ 15:

(TT+CT) vs. CC; OR (95%) = 2.61 (1.05–6.62); p = 0.022

The association of the HLA class II loci and T1D has been long established. The HLA-DRB1*03 and HLA-DRB1*04 alleles are the major contributors to T1D susceptibility. No significant difference in the *PTPN22 *1858T allele frequencies was found between carriers and non carriers of those alleles in the diabetes cohort (DRB1*03 and/or DRB1*04 patients, carriers (61/287) vs. non-carriers (3/29), OR = 2.34; p = 0.16). It has been reported that HLA DR3/DR4 genotype confers the strongest genetic risk for T1D, however, in our Spanish cohort the highest risk genotype for T1D is DRB1*03/DRB1*03 (RR = 22.19 vs. RR = 15.11 for DRB1*03/DRB1*03 and DRB1*03/DRB1*04, respectively; considering RR = 1 subjects negatives for these alleles). Therefore, we repeated the analysis of the *PTPN22 *polymorphism in our population stratified by the two abovementioned genotypes and in subjects without both genotypes. The distribution of 1858T allele was similar in any of the three groups of patients carrying these genotypes: 10/96 (10.4%) in DRB1*03/DRB1*03 patients, 22/180 (12.2%) in heterozygous DRB1*03/DRB1*04 and 37/319 (10.4%) in the rest of patients.

## Discussion

In agreement with recent findings, our data showed an association of the *PTPN22 *1858T allele with T1D in the Spanish population (Table 1). Despite the fact that T1D is an autoimmune disease which does not show sex-bias (gender distribution in our Spanish diabetic population: 50.3% women vs. 49.7% men), Kahles et al [20] reported an association of *PTPN22 *1858T allele only in German T1D females. However, we cannot conclude that this susceptibility factor is exclusively associated with female patients, as it has been proposed, because we could not find a significant difference between diabetic female and male patients (Table 1). This result agrees with several studies which do not show a gender-specific effect [12,14,17,18,24]. The female association found by Kahles et al [20] and the male association reported by Herman et al [25] could be due to spurious effects; however, the analysis in our pediatric-onset patients supports a preferential association in T1D females (Table 2). This age- and gender-dependent association could explain previous results, given that the sex-bias is evidenced only in early-onset patients, but the observed association warrants replication in independent populations. Recent studies have revealed that LYP increases phosphatase activity when allele 1858T is present [26]. This gain of function mutant hypothetically suppresses T cell signaling more efficiently and leads to a failure in apoptosis of autoreactive T cells and to an insufficient activity of regulatory T cells [27]. Given than women have higher absolute numbers of CD4 lymphocytes than men and higher levels of Th1 cytokines [28], this would justify that the observed role of the *PTPN22 *polymorphism is mainly evidenced in girls. Furthermore, in the group with disease onset after 15 years old no gender differences could be established and both female and male patients compared with controls showed OR values of 1.70 (Table 2). In summary, the reasons for sex differences in early onset in T1D is a complex process which remains unclear and could mean that there is an interacting factor with PTPN22 yet to be identified, the effect of sex hormones or epigenetic modifications of DNA could explain this gender-dependent association.

In a recent study, Steck et al [24] described a significant association after stratification by HLA-DRB1 genotypes only in the non-DRB1*03/04 subgroup. However, as they recognize, the lack of association in the high-risk HLA-DRB1 genotype was probably due to compromised statistical power. Moreover, their linear regression analyses did not reveal interaction between *PTPN22 *genotypes and the aforementioned HLA genotype; therefore, they suggest that *PTPN22 *influences T1D risk in all HLA subgroups. The stratification by HLA-DRB1 alleles in our cohort showed no different frequency of the *PTPN22 *1858T allele in any of the patient subgroups. These data suggest that the susceptibility conferred by the *PTPN22 *gene is an independent factor of the HLA effect and patients carrying the *PTPN22 *1858T allele have an increased risk for T1D development independent of this important genetic factor implicated in the disease.

The proposed *PTPN22 *model that allows for more autoreactive T cells to survive and escape into circulation could also determine an earlier age at disease onset; however, no differences were found between patients younger and older than 15 years old.

## Conclusion

In conclusion, we described for the first time in a Spanish population the association of the *PTPN22 *variant with T1D risk. It seems to be a susceptibility factor for both male and female T1D patients, although in pediatric-onset patients the effect seems to be predominant in diabetic females. The age at onset in our cohort is similar in subjects carrying *PTPN22 *1858T allele and in CC homozygous individuals and the susceptibility effect of this variant is independent from the susceptibility conferred by the main HLA-DRB1 alleles associated with T1D.

## Abbreviations

CTLA4 Cytotoxic T-Lymphocyte-Associated protein 4

HLA Human Leukocyte Antigen

LYP Lymphoid Tyrosine Phosphatase

MHC Major Histocompatibility Complex

PTPN22 Protein Tyrosine Phosphatase N22

SNP Single Nucleotide Polymorphism

T1D Type 1 Diabetes

## Competing interests

The authors declare that they have no competing interests.

## Authors' contributions

JLS carried out the genotyping of the patients and a great part of the controls, participated in the statistical analysis and drafted the manuscript. AM carried out a part of the genotyping of control samples and participated in the statistical analysis. HdlC made the diagnosis and collaborated in collection of samples. MAF participated in the genotyping and collection of samples. MFA participated in the coordination of the study and helped to collect the DNA samples. EgdlC coordinated the study and critically revised the manuscript. EU conceived of the study, participated in the statistical analysis and completed the writing of the manuscript.

All authors read and approved the final manuscript.

## Pre-publication history

The pre-publication history for this paper can be accessed here:


